# Total Bilirubin Level is Associated with the Risk of Left Atrial Appendage Thrombosis in Patients with Non-Valvular Atrial Fibrillation

**DOI:** 10.5334/gh.1177

**Published:** 2022-12-22

**Authors:** Weihao Meng, Leigang Wang, Hongxuan Fan, Shaobin Mao, Xiaosu Song, Zhijun Zhang, Haixiong Wang, Bin Liang

**Affiliations:** 1 Shanxi Medical University, Taiyuan, China; 2 Department of Cardiology, Linfen Central Hospital, Linfen, China; 3 Department of Cardiology, The Second Hospital of Shanxi Medical University, Taiyuan, China; 4 Department of Cardiology, Bethune Hospital, Taiyuan, China; 5 Department of Cardiology, Shanxi Cardiovascular Hospital, Taiyuan, China

**Keywords:** non-valvular atrial fibrillation, total bilirubin, left atrial appendage thrombosis

## Abstract

**Objectives:**

There are some evidence suggesting that total bilirubin (TBIL) appears to be associated with stroke in patients with nonvalvular atrial fibrillation (NVAF). The left atrial appendage (LAA) is the most common orgin of thrombus in patients with NVAF. The purpose of this study was to assess a possible relationship between plasma TBIL levels and LAA thrombus in NVAF patients.

**Methods:**

We retrospectively screened 459 consecutive hospitalized patients with NVAF at three AF centers, who underwent transesophageal echocardiography or cardiac CT. According to the examination results, the patients were divided into either the LAA thrombosis group (41 cases) or the no LAA thrombosis group (418 cases). Independent sample t test, Mann-Whitney U-test and chi-square test were used to compare and analyze the general clinical data of the two groups. Multivariate Logistic regression was used to analyze whether TBIL was a risk factor for LAA thrombosis in patients with NVAF. Pearson correlation analysis was used to explore the correlation between TBIL and other influencing factors. The predictive value of TBIL for LAA thrombosis in patients with NVAF was evaluated by ROC curve.

**Results:**

A total of 459 patients were enrolled in this study. Compared with the group without LAA thrombosis, the level of TBIL in LAA thrombosis group was significantly increased (21.34 ± 9.34 umol/L vs. 13.98 ± 4.25 umol/L, *P* < 0.001). Multivariate logistic regression showed that TBIL level was a risk factor for LAA thrombosis (*OR*, 1.229; 95% *CI*, 1.122~1.345; *P* < 0.001). The AUC of the ROC curve is 0.801 (95% *CI*, 0.725~0.877; *P* < 0.001). At 17.4 umol/L of TBIL, the patient may have LAA thrombosis (sensitivity 73.2%; specificity 82.1%).

**Conclusions:**

In patients with NVAF, TBIL level is positively associated with LAA thrombosis, and TBIL level may be an index reflecting LAA thrombosis.

## 1 Introduction

AF is the most common tachyarrhythmia in clinical practice and is one of the major risk factors for thromboembolic events. Thromboembolism is the most serious complication that increases morbidity and mortality in patients with AF. NVAF may increase the risk of ischemic stroke by 4–5 times [1]. Nearly 100% of ischemic stroke emboli in patients with NVAF originated from the LAA [2]. In clinical practice, the CHA2DS2-VASC score has been widely used to predict the risk of embolic events in patients with NVAF. However, the CHA2DS2-VASC score seems to be more appropriate for high-risk stroke patients, and there are still some patients with low scores who still have LAA thrombosis. Transesophageal echocardiography and cardiac CT can be used to detect the formation of LAA thrombosis, but both of them have certain risks and contraindications, so they are not suitable for all patients with AF. Therefore, it is crucial to find reliable alternative biomarkers. TBIL is an objective index for quantitative detection of hepatobiliary diseases and hemolytic diseases, and has been a popular research topic in recent years. At present, a large number of studies have found that TBIL is associated with the occurrence of AF, the recurrence of AF after catheter ablation, and the occurrence of ischemic stroke in patients with AF, and its mechanism may be related to inflammation [3,4,5,6]. However, the relationship between TBIL and LAA thrombosis in patients with NVAF is unknown. Therefore, we erolled NVAF patients with LAA thrombus and no No LAA thrombus. Clinical data were analyzed, the relationship between TBIL and LAA thrombosis and its predictive value in patients with NVAF was explored.

## 2 Methods

### 2.1 Patient population

Four hundred fifty-nine Patients with AF admitted to the Second Hospital of Shanxi Medical University, Shanxi Bethune Hospital and Shanxi Cardiovascular Hospital from January 2019 to January 2021 were retrospectively evaluated. Inclusion criteria: AF patients were diagnosed by the result of electrocardiogram or dynamic electrocardiogram. All patients received transesophageal echocardiography or cardiac CT. Transesophageal echocardiography were performed in 298 patients and Cardiac CT were performed in 161 patients. The following criteria was excluded: valvular heart disease, acute coronary syndrome, history of hemorrhagic stroke, viral hepatitis, severe renal insufficiency, severe infection, malignant tumor, severe connective tissue disease, and contrast agent allergy. General clinical data of selected patients were collected through electronic medical records, including general information such as age, gender, BMI, past history, personal history, Cardiac parameters, CHA2DS2-VASC score and HAS-BLED score. Fasting cubital vein blood (3 mL) was collected in the morning of the day after admission and placed in anticoagulant or procoagulant tubes for examination. TBIL, alanine aminotrans-ferase (ALT), creatinine (Cr), and other biochemical indicators were detected using enzyme-linked immunosorbent assay. Red blood cell volume distribution width (RDW) was detected using fully automated blood cell analyzer. D-dimer (D-Di) was detected using immunoturbidimetry assay. AF patients were divided into paroxysmal AF group and nonparoxysmal AF group according to ESC Atrial Fibrillation Management Guidelines 2020 [7]. According to the results of transesophageal echocardiography and cardiac CT, LAA thrombosis was determined. Patients were divided into LAA thrombus group (n = 41) and No LAA thrombus group (n = 418). The protocol was approved by the institutional ethics review committee.

### 2.2 Transesophageal echocardiography (TEE)

The transesophageal echocardiography was performed using ultrasound apparatus, water was forbidden and fasting for 8 hours. The pharynx was subjected to local anesthesia with lidocaine gel. The ultrasound probe was inserted into the esophagus to the middle esophageal level, and the position of the probe was adjusted at 0 ~ 180° to continuously evaluate the left atrium (LA) and LAA. The results were interpreted by two experienced ultrasound physicians according to the real-time ultrasound images. LAA thrombosis was defined as a clump-like echo-dense area with well-defined edges and homogeneous internal echoes in at least two or more cross-sectional images.

### 2.3 Cardiac CT

The region of interest was placed in the LA for a two-phase scan by CT scanner. A high pressure syringe was used to inject 65–70 ml ioprolamide 370 through the forearm vein at 4.5–5 ml/s for the first stage of scanning. The second stage of scanning was carried out 7 ~ 8 S after the concentration reached the peak. The images after scanning were based on cross-sectional images. The images were reconstructed by means of volume reconstruction, maximum density projection and multi-plane reconstruction, and the results were interpreted by two experienced radiologists. LAA thrombosis was defined as a filling defect at the internal fixation site of the LAA in both phases after the injection of contrast agent.

### 2.4 Statistical analysis

SPSS20.0 statistical software was used for data analysis (Chicago, IL, USA). All continuous variables were expressed as the mean ± SD. The Independent sample t test and the Mann-Whitney U-test were used to compare parametric and nonparametric continuous variables, respectively. The chi-square test was used to compare categorical variables. Multivariate Logistic regression were used to analyze whether TBIL was a risk factor for LAA thrombosis in patients with NVAF. Pearson correlation analysis was used to explore the correlation between TBIL and other influencing factors. The ROC curve and the area under the curve (AUC) were used to explore the relationship between TBIL level and LAA thrombosis. The maximum value of the sum of sensitivity and specificity on the ROC curve was taken as the optimal bilirubin boundary value that could form LAA thrombosis, and *P* < 0.05 was considered to be statistically significant.

## 3 Results

### 3.1 Baseline data of patients

A total of 459 patients with NVAF were enrolled in this study, including 193 females (42.0%), with a mean age of 63.1 ± 10.8 years, and 224 patients (48.8%) over 65 years, 299 patients (65.1%) were diagnosed of paroxysmal AF. There were 265 cases of hypertension (57.7%), 98 cases of diabetes mellitus (21.3%), 108 cases of coronary heart disease (23.5%), 9 cases of history of myocardial infarction (1.9%), and 81 cases had a history of stroke (17.6%). Among the total patients, 15 patients (9.3%) were diagnosed of LAA thrombosis by cardiac CT, and 26 patients (8.7%) by TEE. 418 patients (91.1%) were enrolled in the No LAA thrombus group and 41 patients (8.9%) in the LAA thrombus group.

Table 1 summarizes the demographic characteristics and clinical data comparison of the study population. The LAA thrombosis group had a higher percentage of nonparoxystal AF compared with the No LAA thrombosis group (87.8% vs. 29.6%, *P* < 0.001), a larger RDW (13.88 ± 1.31% vs. 13.43 ± 2.01%, *P* = 0.003), a larger D-Di (350.16 ± 573.10 ng/ml vs. 186.78 ± 294.59 ng/ml, *P* = 0.026), a larger left atrium diameter (LAD) (47.72 ± 4.21 mm vs. 42.85 ± 6.14 mm, *P* < 0.001), and a higher CHA2DS2-VASc score (3.88 ± 1.24 vs. 2.17 ± 1.53, *P* < 0.001). Compared with the group without LAA thrombosis, the LAA thrombosis group had lower left ventricular ejection fraction (LVEF) (58.88 ± 12.01% vs. 63.5 ± 5.76%, *P* = 0.020)and lower left ventricular fractional shortening (LVFS) (31.98 ± 7.86% vs. 34.66 ± 4.64%, *P* = 0.046).

**Table 1:** Baseline characteristics of patients with or without LAA thrombus.


PARAMETER	NO THROMBUS (N = 418)	THROMBUS (N = 41)	*P* VALUE

	0.096	756	

Age, years	63.15 ± 11.01	62.95 ± 8.63	0.756

Female, n (%)	174 (41.6)	19 (46.3)	0.559

BMI, kg/m^2^	25.18 ± 3.83	24.72 ± 3.07	0.682

Nonparoxymal AF, n (%)	124 (29.6)	36 (87.8)	<0.001

Hypertension, n (%)	240 (57.4)	25 (60.9)	0.660

Diabetes, n (%)	92 (22.0)	6 (14.6)	0.271

Coronary artery disease, n (%)	102 (24.4)	6 (14.6)	0.159

Old myocardial infarction, n (%)	8 (1.9)	1 (2.4)	0.817

Previous stroke/TIA, n (%)	70 (16.7)	11 (26.8)	0.106

Smoking, n (%)	130 (31.1)	11 (26.8)	0.572

TBIL, umol/L	13.98 ± 4.25	21.34 ± 9.34	<0.001

ALT, U/L	24.01 ± 17.52	25.42 ± 17.83	0.492

RDW,%	13.43 ± 2.01	13.88 ± 1.31	0.003

Cr, umol/L	72.91 ± 17.52	73.79 ± 15.97	0.738

D-Di, ng/ml	186.78 ± 294.59	350.16 ± 573.10	0.026

LAD, mm	37.59 ± 5.92	42.85 ± 6.14	<0.001

LVDd, mm	47.72 ± 4.21	49.39 ± 7.42	0.261

LVEF,%	63.5 ± 5.76	58.88 ± 12.01	0.020

LVFS,%	34.66 ± 4.64	31.98 ± 7.86	0.046

CHA_2_DS_2_-VASc	2.17 ± 1.53	3.88 ± 1.24	<0.001

HAS-BLED	0.94 ± 0.99	1.02 ± 0.96	0.485


AF: atrial fibrillation; TBIL: Total bilirubin; ALT: Alanine aminotransferase; RDW: Red blood cell volume distribution width; Cr: Creatinine; D-Di: D-dimer; LAD: Left atrium diameter; LVDd: Left ventricular end diastolic diameter; LVEF: Left ventricular ejection fraction; LVFS: Left ventricular fractional shortening.

### 3.2 Relationship between TBIL and LAA thrombosis in patients with NVAF

**Figure 1:**
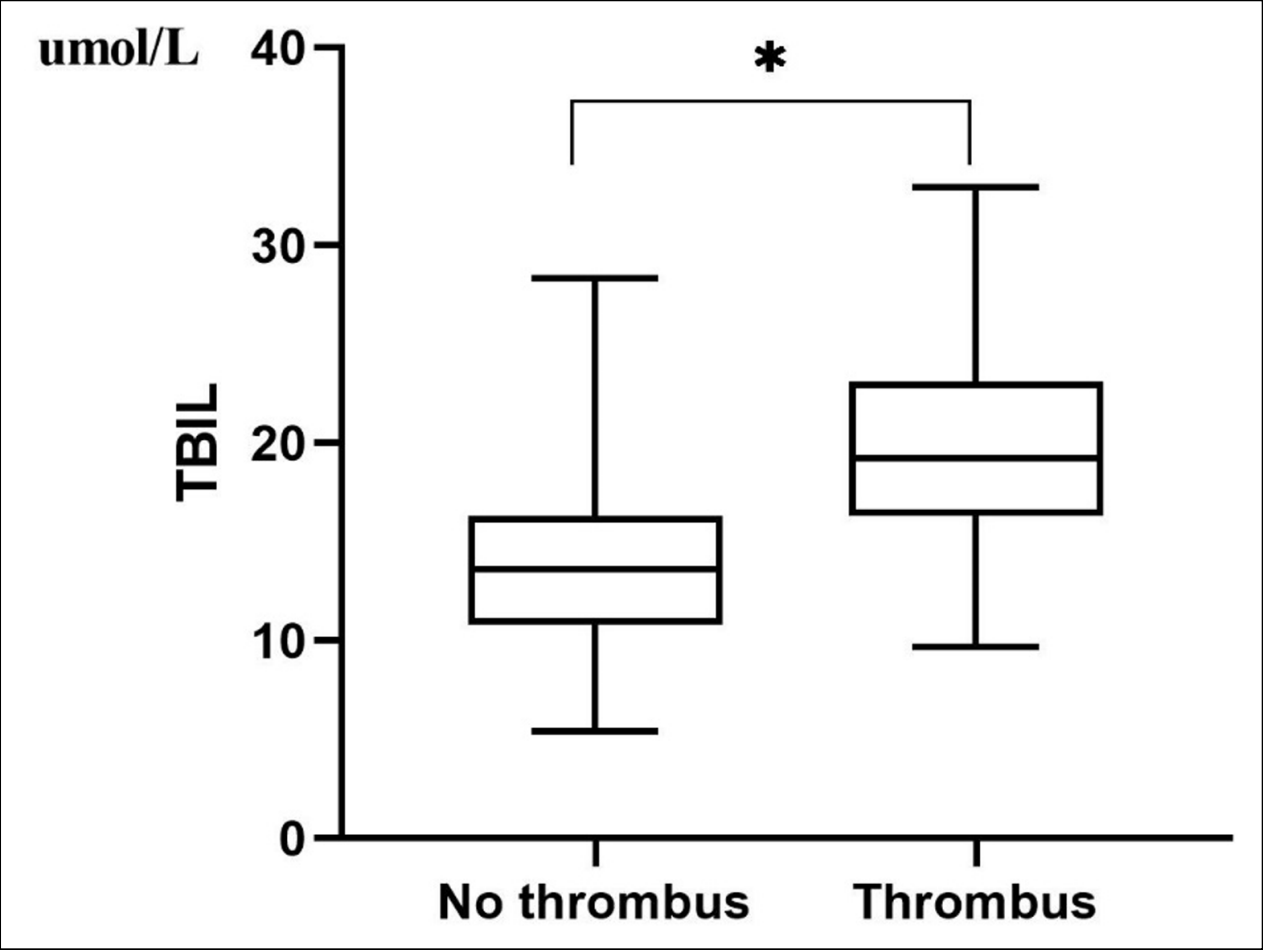
Plasma TBIL levels in patients with LAA thrombus compared with those without LAA thrombus. (‘*’ with statistical significance).

As can be seen from Table 1 and Figure 1, serum TBIL levels were significantly higher in NVAF patients with LAA thrombosis than without LAA thrombosis (21.34 ± 9.34 umol/L vs. 13.98 ± 4.25 umol/L, *P* < 0.001). Serum TBIL levels in NVAF patients with LAA thrombosis were negatively correlated with the size of LVEF (r = –0.327, *P* = 0.031)(Table 3).

According to the results in Table 1, nonparoxystal AF, TBIL, RDW, D-Di, LAD, LVEF, LVFS and CHA2DS2-VASC were influencing factors, and taking these influencing factors into multivariate logistic regression analysis, the result showed that RDW, D-Di, LAD and LVFS were not risk factors in NVAF patients with LAA thrombosis, whereas nonparoxystal AF (OR, 17.073; 95% CI, 4.633–62.914; *P* < 0.001), TBIL (OR, 1.229; 95% CI, 1.122–1.345; *P* < 0.001) and CHA2DS2-VASC (OR, 1.974; 95% CI, 1.459–2.670; *P* < 0.001) were still risk factors in NVAF patients with LAA thrombosis. LVEF (OR, 0.873; 95% CI, 0.770–0.989; *P* = 0.033)was a protective factor in NVAF patients with LAA thrombosis (Table 2).

**Table 2:** Multivariate logistic regression analysis of LAA thrombus.


VARIABLE	OR	95%CI	*P* VALUE

Nonparoxymal AF	17.073	4.633–62.914	<0.001

TBIL	1.229	1.122–1.345	<0.001

RDW	1.063	0.837–1.351	0.614

D-Di	1.001	1.000–1.002	0.133

LAD	1.017	0.940–1.099	0.678

LVEF	0.873	0.770–0.989	0.033

LVFS	1.161	0.984–1.369	0.076

CHA_2_DS_2_-VASc	1.974	1.459–2.670	<0.001


AF: atrial fibrillation; TBIL: Total bilirubin; RDW: Red blood cell volume distribution width; D-Di: D-dimer; LAD: Left atrium diameter; LVEF: Left ventricular ejection fraction; LVFS: Left ventricular fractional shortening.

**Table 3:** Pearson correlation analysis between TBIL and its clinical data in AF patients with LAA thrombus.


PARAMETER	*r*	*P* VALUE

RDW	0.207	0.177

D-Di	0.150	0.331

LAD	0.124	0.422

LVEF	–0.327	0.031

LVFS	–0.295	0.052

CHA_2_DS_2_-VASc	–0.157	0.308


TBIL: Total bilirubin; RDW: Red blood cell volume distribution width; D-Di: D-dimer; LAD: Left atrium diameter; LVEF: Left ventricular ejection fraction; LVFS: Left ventricular fractional shortening.

### 3.3 Resolution of TBIL by ROC curve in patients with or without LAA thrombosis

**Figure 2:**
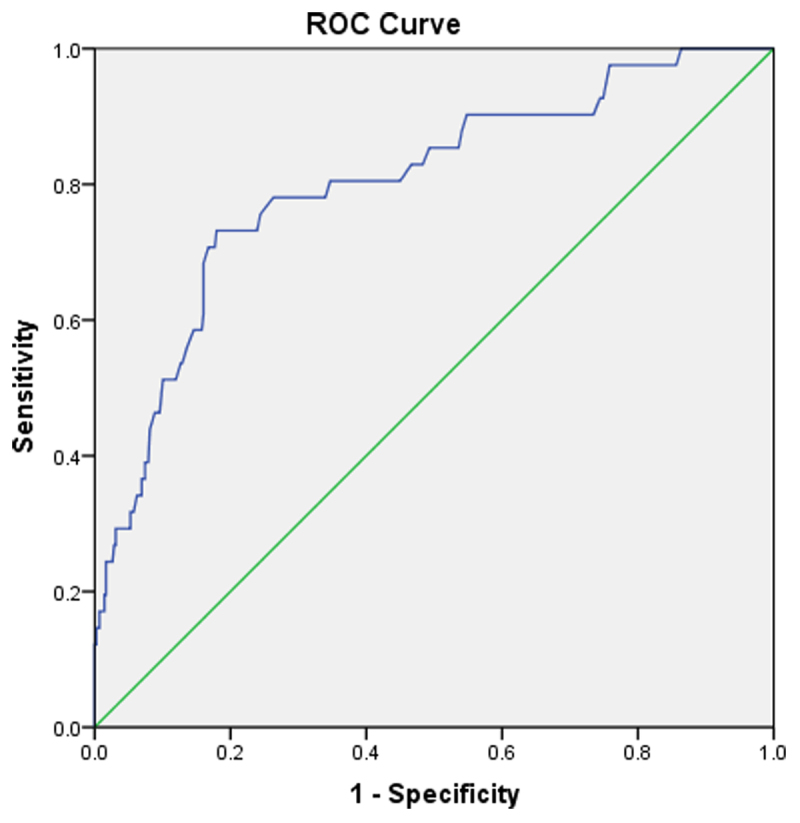
ROC curve for TBIL in predicting LAA thrombosis.

Based on the ROC curve analysis in Figure 2, TBIL shows a predictive value of 0.801 (95%CI, 0.725 ~ 0.877; *P* < 0.001). The optimal cutoff level of TBIL for predicting LAA thrombosis was 17.4 umol/L, with a sensitivity of 73.2% and a specificity of 82.1%.

## 4 Discussion

LAA, a tubular diverticulum of the LA, is the most common origin of AF thrombosis due to its unique anatomical structure. LAA thrombosis is a major cause of thromboembolic events in patients with AF and increases the risk of death. Therefore, it is of great clinical significance to study the risk factors of LAA thrombosis for the treatment of AF and prevention of thromboembolic events. However, the incidence of LAA thrombosis in patients with AF varies due to different study regions and living habits. Zhan et al. [8] showed that the incidence of LAA thrombosis in patients with AF who did not receive anticoagulation or non-standard anticoagulation therapy was 5–27%. Our study showed that the incidence of LAA thrombosis in patients with AF was about 8.9%, which is similar to the previous studies. Habara et al. [9] showed that 925 patients with NVAF were enrolled in their study, with a LAA thrombosis incidence of 9.0%. These findings suggest that LAA thrombosis is an important concern in the treatment of patients with AF. Furthermore, in clinical practice, TEE and cardiac CT were used to diagnose LAA thrombosis in AF patients, although cardiac CT is less accurate than TEE. In our study, the diagnostic rate of cardiac CT is 9.3%, which is slightly higher than that of TEE, which is 8.7%. and this result may affect the accuracy of diagnosing LAA thrombosis, and future studies can investigate the difference between TEE and cardiac CT.

Current studies have found that multiple factors are associated with LAA thrombosis, among which the type of AF is an important factor in predicting LAA thrombosis formation in patients with AF. Kapłon-cieślicka et al. [10] found that type of AF was a strong predictor of LAA thrombosis in patients with AF. In addition, GYH et al. [11] showed a higher risk of LAA thrombosis in patients with nonparoxysmal AF compared with patients with paroxysmal AF. Our results show that nonparoxysmal AF is a risk factor for LAA thrombosis in patients with NVAF, which is consistent with these findings. LVEF is an important ultrasonographic indicators reflecting left ventricular function. Our study found that patients with AF with low LVEF was more likely to form LAA thrombosis, which was similar to previous findings [12,13]. In clinical practice, CHA2DS2-VASC score is often used to evaluate the risk of stroke and thromboembolism in patients with NVAF. In this study, 298 patients had an elevated CHA2DS2-VASC score (≥2), of which 41 patients had LAA thrombosis. There were significant differences in CHA2DS2-VASC scores between AF patients with and without LAA thrombosis. These results are consistent with previous findings. Bertaglia et al. [14] shows that CHA2DS2-VASC is a risk factor for LAA thrombosis in NVAF patients.

Bilirubin is the main end product of heme decomposition, which is produced by heme oxygenase to decompose aging red blood cells. It is often used as a diagnostic index of liver and biliary diseases and hemolytic diseases in clinical practice. In recent years, many studies have found that serum TBIL levels are associated with coronary heart disease, heart failure, atrial fibrillation and other cardiovascular diseases [15,16,17]. Kuwabara et al. [3] found that Serum TBIL levels in patients with AF are significantly higher than those in patients without AF. Lin et al. [4] study had obtained similar results also. The possible mechanisms are as follows: ① Oxidative stress and inflammation increase TBIL levels in AF patients [5]; ② In AF patients, hemodynamics is unstable, and heme oxygenase, which is involved in hemoglobin degradation, is activated in the atrium and liver, leading to increased serum TBIL levels [18,19]; ③ AF may change the gene expression and functional profile of rat liver, enhance the interleukin-6 (IL-6) signaling pathway, and lead to the increase of TBIL level [20]. Higher TBIL levels are associated with recurrence of AF after catheter ablation in patients with paroxysmal AF, suggesting that elevated TBIL levels may be an important factor in the onset and maintenance of AF [5]. In addition, TBIL is significantly correlated with ischemic stroke. Lin et al. [4] found that elevated serum TBIL level is a risk factor for cardiac stroke and an important indicator to distinguish cardiac stroke from other types of stroke. Liu et al. [6] found that TBIL level in NVAF patients with ischemic stroke was significantly higher than that in patients without ischemic stroke, indicating that the increase of TBIL level may be one of the important reasons for the increase of stroke rate in NVAF patients. To date, there is no evidence of an association between TBIL and LAA thrombosis in AF patients. Our results support the idea that TBIL may be involved in LAA thrombosis formation during AF. To our knowledge, this is the first study to elaborate the relationship between TBIL and LAA thrombosis. In this study, we investigated the relationship between serum TBIL levels and LAA thrombosis in 459 patients with NVAF using a multicenter database. The main results were as follows: ① TBIL levels in NVAF patients with LAA thrombosis were significantly higher than those in NVAF patients without LAA thrombosis; ② TBIL level is positively associated with LAA thrombosis in patients with NVAF; ③ TBIL is a risk factor for LAA thrombosis in patients with NVAF; ④ TBIL may be an emerging index reflecting LAA thrombosis that differs from previous evaluation standards. However, the mechanism of how TBIL influences LAA thrombosis formation is still unclear. The possible mechanism may be when AF developed, atrial rhythm and effective myocardial contraction were impaired. Blood flow became slow, hypostasis and vortex were formed, which promoted thrombosis. The filling of left ventricle was decreased, which reduced left cardiac output. Peripheral organ hypoperfusion caused liver ischemia, and the level of TBIL was finally increased. As our study found that TBIL is negatively correlated with LVEF. TBIL level may be associated with left atrial blood stasis. Elevated TBIL levels may indicate LAA thrombosis formation. In addition, AF can induce inflammation, which plays an important role in endothelial damage and blood hypercoagulation. Inflammation can cause atrial endothelial injury, promote platelet adhesion and aggregation, and LAA thrombosis formation. Endothelial injury can also lead to reduced nitric oxide synthesis, resulting in imbalance of blood coagulation and fibrinolysis system, which can result in hypercoagulability of blood and further promoting LAA thrombosis formation [21,22,23]. As an endogenous anti-inflammatory molecule, the level of TBIL is closely related to inflammation. TBIL level may reflect endothelial damage and blood hypercoagulability, and elevated TBIL level may indicate the formation of LAA thrombosis.

### Limitations

This study has several limitations. Firstly, this multi-center retrospective study has a small sample size, and all the patients are from inpatients, which has certain selection bias and cannot truly reflect the whole population of AF. Secondly, this study was a retrospective study, some AF centers failed to use TEE to diagnose LAA thrombosis, instead cardiac CT, although TEE is the gold standard for diagnosing LAA thrombosis, and some laboratory indicators related to LAA thrombosis formation of patients were not done or different detection methods for relevant indicators were used in different atrial fibrillation centers, leading to different test results, such as uric acid, BNP, inflammatory indicators and LAA velocity, and so forth, so the variables were not included in the study. Finally, in clinical practice, TBIL, as a convenient and effective test indicator, can predict the occurrence of atrial fibrillation and LAA thrombosis to some extent, but the specific mechanism of TBIL in the occurrence of atrial fibrillation and LAA thrombosis formation is still unclear, and further research and exploration are needed in the future.

## Conclusions

In patients with NVAF, TBIL level is positively associated with LAA thrombosis, and TBIL may be an index reflecting LAA thrombosis.
